# Personalized Cup Positioning Guides Improved Cup Positioning and Hip Ranges of Motion in Robotic Assisted Total Hip Arthroplasty

**DOI:** 10.3389/fbioe.2020.00988

**Published:** 2020-08-21

**Authors:** Ruoyu Wang, Xiaojing Zheng, Tianze Xu, Song Gong, Shaokai Liu, Lizhi Han, Shuhua Yang, Weihua Xu

**Affiliations:** ^1^Department of Orthopaedics, Union Hospital, Tongji Medical College, Huazhong University of Science and Technology, Wuhan, China; ^2^Department of Pediatrics, The University of North Carolina at Chapel Hill, Chapel Hill, NC, United States; ^3^Department of Biostatistics, The University of North Carolina at Chapel Hill, Chapel Hill, NC, United States; ^4^College of ACES, University of Illinois at Urbana-Champaign, Urbana, IL, United States

**Keywords:** robotic assisted total hip arthroplasty, dislocation, impingement, cup positioning, hip ranges of motion

## Abstract

**Objective:**

Precise hip cup positioning is essential for the prevention of component impingement and dislocation in robotic assisted total hip arthroplasty (THA). Currently, the robotic system uses a mechanical alignment guide (MAG) for cup placement, which is one-size-fits-all, and the optimal cup positioning is controversial. Robotic assisted THA has not used any personalized cup positioning guides. The goal of this study was to identify an optimal guide for cup placement in robotic assisted THA to improve prognosis and life quality after THA.

**Materials and Methods:**

Pelvis and femoral CT data of 47 participants were retrospectively collected for preoperative planning of robotic THA. The universal MAG guide and three personalized guides, including acetabular rim labrum guide (ARLG), transverse acetabular ligament guide (TALG), and ischiatic-pubis line guide (IPLG), were used to pose cups in the acetabulum of each participant. The position of cups was evaluated by inclination and anteversion; the function of hip joints was evaluated by hip ranges of motion, including abduction, adduction, extension, flexion, internal rotation, and external rotation.

**Results:**

In terms of cup positioning, ARLG provided a bigger cup inclination (*p* < 0.0001), while IPLG and TALG provided smaller cup inclination (*p* < 0.001) than MAG; the three personalized guides provided larger cup anteversion (*p* < 0.0001) than MAG. In terms of HROMs, compared with the use of MAG, the use of three personalized guides significantly decreased abduction (*p* < 0.0001), extension (*p* < 0.0001), and external rotation (*p* < 0.0001), but increased significantly flexion (*p* < 0.0001) and internal rotation (*p* < 0.0001); the use of ARLG significantly reduced adduction (*p* < 0.0001), but the use of IPLG and TALG increased adduction (*p* < 0.0001).

**Conclusion:**

Compared with MAG, personalized guides provided greater flexion and internal rotation, which may reduce the risk of posterior dislocation. Among the three personalized guides, IPLG is the most reliable one for the preoperative planning of robotic assisted THA.

## Introduction

Total hip arthroplasty (THA) is the most effective treatment for the end stages of hip diseases (Pivec et al., 2012). Since the 1980s, the use of robotics in assisting the surgical procedure was gradually introduced to THA. Many robotic systems were developed to improve the precision of surgery (Illgen et al., 2017). Robotic assisted THA is considered safer and more accurate than traditional THA with fewer complications, less trauma, and attainable superior long-term clinical outcomes. Optimal component positioning is one surgeon-controlled factor that plays a significant role in preventing complications including hip dislocations, accelerated bearing wear, poor biomechanics, leg length discrepancy, and revision surgery (Abdel et al., 2015; De Martino et al., 2017; Seagrave et al., 2017).

Preoperative planning is the most important step in robotic assisted THA to determine the accuracy of components. One of the most important factors indicating component impingement after THA (Peter et al., 2015) is the hip range of motion (HROM), which consists of six parameters, including abduction, adduction, extension, flexion, internal rotation, and external rotation. Reduction of flexion, adduction, and internal rotation indicates an impingement between the anterior acetabular component (cup) and femoral component (stem), which may lead to posterior dislocation; meanwhile, reduction of extension and external rotation indicates an impingement between posterior cup and stem, which may lead to anterior dislocation (Mccarthy et al., 2016). The preoperative planning of the mainstream robot-assisted systems (MAKO and ROBODOC) uses CT data to reconstruct pelvis and hip joints and perform simulated surgery (Tarwala and Dorr, 2011; Jacofsky and Allen, 2016). Previous studies evidenced that the preoperative planning based on simulated surgery could accurately predict the position of the prosthesis after THA (Sato et al., 2000; Scifert et al., 2001; Won et al., 2013). Several studies (Stansfield et al., 2003; Fischer et al., 2018) have compared the simulated HROMs in preoperative planning and the measured post-THA HROMs and indicated that estimated HROMs in preoperative planning could be used as a surrogate to predict the HROMs after THA. Preoperative planning has several steps, taking MAKO as an example, including (1) importing CT data and reconstructing pelvis and hips, (2) determining the reference planes, (3) planning cup positioning, (4) planning stem positioning, and (5) estimating the effect of the preoperative plan (Qin et al., 2018).

Multiple factors may influence HROM, including positioning, height, depth, and diameter of the cup, as well as head size, depth, offset, and neck length of the stem. Among these factors, cup positioning is the most critical factor (Jones, 2015; Seagrave et al., 2017). Accurate cup positioning is essential to avoid component impingement and dislocation. Cup positioning consists of two parameters: inclination and anteversion. Inclination is defined as the angle between the patient’s sagittal plane and the axis of the cup. Anteversion is defined as the angle between the patient’s vertical axis and the axis of the cup projected on the sagittal plane of the patient. Universal and personalized guides have been reported to improve the accuracy of cup positioning in traditional THA (Digioia et al., 2002; Beverland et al., 2016; Rutherford et al., 2018). Mechanical alignment guide (MAG) is the most widely used universal guide (Rutherford et al., 2018), due to its easy use. MAG assumes that the operating table parallels with the sagittal plane, and the long axis of the operating table parallels the vertical axis of the patient; however, any changes in the relative position of the patient and the operating table may influence the accuracy of cup positioning. The use of MAG, reported by Bosker et al. (2007), caused inaccuracy in both cup inclination and anteversion MAG caused an average deviation of 6.3° in cup inclination and an average 5.7° in anteversion. Assuming that the goal of cup poisoning was the inclination of 40° and anteversion of 15°, there were deviations of 15.25% in inclination and 38% in anteversion. Transverse acetabular ligament guide (TALG) uses transverse acetabular ligament to provide a personalized cup position and to reduce dislocation rate (Archbold et al., 2006). Nevertheless, the absence, calcification, and destruction of the transverse acetabular ligament may disable the guide for positioning. Acetabular rim labrum guide (ARLG) uses acetabular rim and labrum to provide personalized cup positioning (Higgins et al., 2014). ARLG works well when lesion of the pelvis is mild, but it is unstable when acetabulum suffers severe lesions, such as late-stage femoral head necrosis and developmental dysplasia of the hip. Ischiatic-pubis line guide (IPLG) uses three peri-acetabulum anatomic landmarks to provide personalized cup positioning (Sotereanos et al., 2006), but the over-exposure of muscles around acetabulum may cause additional damage.

Currently, mainstream robotic assisted systems (including MAKO and ROBODOC) use MAG to determine the positioning of cups. MAG is one-size-fits-all, thus potentially leading to controversial optimal positioning (Abdel et al., 2015; Tezuka et al., 2019). Personalized cup placement guides have not been used in robotic assisted THA. There are various approaches to THA. The most widely used THA in the world is the posterior approach (James et al., 2010). This study aimed to identify an optimal cup positioning guide for robotic assisted THA, by comparing the effects of MAG and three personalized guides (TALG, ARLG, IPLG) on cup positioning and HROMs in preoperative planning of robotic assisted THA, under the posterior approach.

## Materials and Methods

### Data Collection

Retrospective computer tomography angiography (CT) from all patients in 2016 was retrieved from the imaging database of Union Hospital in Wuhan, China. The inclusion criteria followed two principles: (1) CT angiography should contain bilateral femurs and whole pelvis, and (2) the participants are older than 18 years old. The study also excluded participants with orthopedic diseases including fracture, deformity osteoarthritis (OA), dysplasia of the hip (DDH), osteonecrosis of the femoral head (ONFH), tumor, and previous orthopedic operation. Moreover, the study excluded participants whose TAL and labrum did not reveal under CT.

Amongst 90 participants who met the criteria, this study excludes another 24 participants due to DDH, ONFH, OA, and previous orthopedic operations, and 19 participants because either TAL or labrum could not be observed under CT. Eventually, CT data from 47 participants, saved in DICOM format, was used for further analysis.

Ethics Committee of Tongji Medical College, Huazhong University of Science and Technology, approved this study. This study was a retrospective study, and no patients received additional X-rays because of this study. Ethics Committee approved a waiver of informed consent for this retrospective study of Tongji Medical College.

### Preoperative Planning

The preoperative planning included regeneration of pelvis and hips from CT data, determination of reference planes, cup positioning planning, stem positioning planning, and cup positioning evaluation.

#### CT Data Regeneration and Determination of Reference Planes

CT data of lower extremities were loaded into Mimics Research 17.0 (Materialise Inc., Belgium) to regenerate whole pelvises and bilateral femurs by CT bone segmentation for THA simulation. A reliable 3D pelvic coordinate system was determined by the anterior pelvic plane, mid-sagittal plane, and transverse plane with the method by Dai (Zhang et al., 2017). Briefly, anterior pelvic plane (equivalent to the coronal plane) was determined by the bilateral anterior superior spine and pubic symphysis; the sacral spinous process determined mid-sagittal plane (equivalent to sagittal plane), the midpoint of the bilateral anterior superior spine, and pubic symphysis. The transverse plane was determined by both coronal and sagittal planes and was perpendicular to the tow planes.

#### Cup Positioning Planning

The cup planning was determined by four parameters: positioning, height, depth, and diameter. We referred to the method proposed by Shen for the preoperative planning of the cup (Zeng et al., 2014). We evaluated the effects of four guides – MAG, TALG, ARLG, and IPLG – on positioning and HROMs while keeping the other parameters – including the cup, height, depth, and diameter – the same to avoid their confounding effects.

**FIGURE 1:**
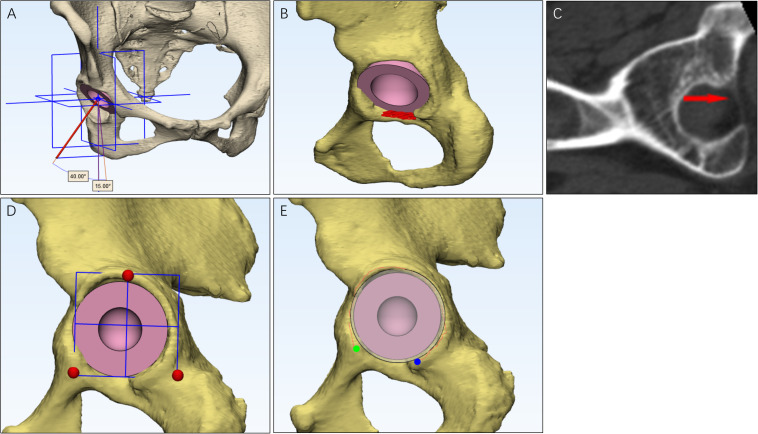
The methods to use the four cup positioning guides. For the mechanical alignment guide **(A)**, inclination was the angle between the cup axis and the sagittal plane, and the anteversion was the angle between the longitude axis and the cup axis that was projected to the sagittal plane. For transverse acetabular ligament guide **(B)**, the face of the cup was placed parallel to the transverse acetabular ligament, and the face was attached to the inner edge of the transverse acetabular ligament, which could be observed in CT **(C)**. When using the ischiadic-pubis line guide **(D)**, the face of the cup was paralleled to a plane that was consisted of three points, including the lowest point of the acetabular sulcus of the ischium, the prominence of the superior pelvic ramus, and the most superior point of the acetabular rim. When using acetabular rim labrum guide **(E)**, the face of the cup was paralleled to the plane that is fit to the labrum around the acetabular rim.

When applying MAG, the angle between the cup axis and the sagittal plane set at 40 degrees and the angle between the cup axis projected to the sagittal plane and vertical axis set at 15 degrees (Figure 1A). When using TALG, the face of the cup was placed parallel and attached to the inner edge of the transverse acetabular ligament (Figures 1B,C; Archbold et al., 2006). When using IPLG, the face of the cup was paralleled with a plane consisting of three points, including lowest point of the acetabular sulcus of the ischium, the prominence of the superior pelvic ramus, and the most superior point of the acetabular rim (Figure 1D; Sotereanos et al., 2006). When using ARLG, the face of the cup was paralleled with the plane that fitted to the labrum around the acetabular rim (Figure 1E).

The diameter of the femoral head determined the size of the acetabular prosthesis. The acetabular prosthesis was placed close to the acetabular fovea with the original height of the rotation center (Daines and Dennis, 2012; Fukui et al., 2013).

#### Stem Positioning Planning

The stem planning included four parameters: head size, depth, offset, and neck length. We referred to the method proposed by Akiyama for the preoperative planning of stem (Akiyama and Shibuya, 2018). All four parameters were kept the same to avoid confounding factors. A stem of M/L Taper (Zimmer, United States) was inserted into the femoral shaft. The femoral anteversion of femoral prothesis was consistent with the original femoral anteversion of each participant. Femoral heads with 28 mm diameters were used for all participants. Three kinds of neck, −3.5 mm, 0 mm, and + 3.5 mm, were prepared to keep the length of the femoral net consistent with original ones.

#### Evaluation of Cup Positioning

The two parameters of cup positioning, including inclination and anteversion, were measured with the Murray method (Murray, 1993).

### HROMs Prediction

Six parameters of HROMs, abduction, adduction, extension, flexion, internal rotation, and external rotation, were measured.

**FIGURE 2:**
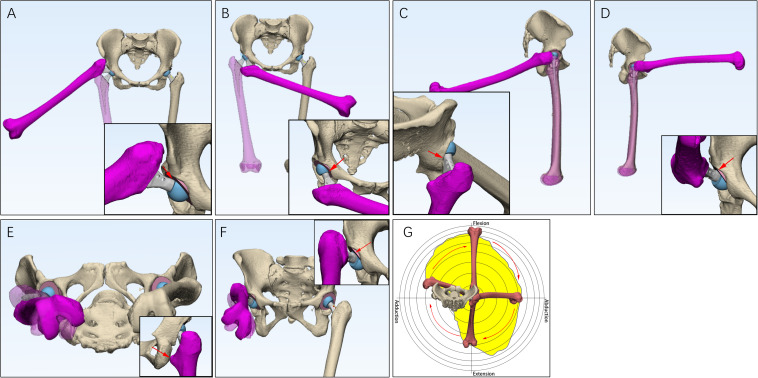
The measurement of hip ranges of motion (HROMs) and boundary map. Six parameters of HROMs were measured. The femur was rotated outward and inward in the coronal plane to test abduction **(A)** and adduction **(B)**, respectively. The femur was rotated backward and forward in the sagittal plane to test extension **(C)** and flexion **(D)**, respectively. The femur was rotated around inward and outward around the sagittal axis to test internal rotation **(E)** and external rotation **(F)**, respectively. When measuring the boundary map, the femur was manipulated into performing circumduction **(G)**.

The procedure rotated femur outward and inward in the coronal plane to test abduction and adduction, respectively (Figures 2A,B); rotated femur forward and backward in the sagittal plane to test flexion and extension, respectively (Figures 2C,D); rotated femur inward and outward around the sagittal axis to test internal rotation and external rotation, respectively (Figures 2E,F). The point in which cup-neck impingement occurred represented the maximum range of motion.

A boundary map of HROMs showed a combined effect of abduction, adduction, extension, and flexion. We referred to the method proposed by Griffin for testing boundary map (Turley et al., 2013). Briefly, the femur was circumferentially rotated. The point at which cup-neck impingement occurred was plotted on a two-dimensional plot, which permitted range of motion to be represented graphically as a continuum (Figure 2G; Jie et al., 2016).

### Data Analysis

Parameters of cup positioning and HROMs were tested for normal distribution by using the Shapiro-Wilk normality tests. Intraclass correlation coefficients (ICCs) were calculated to evaluate the measurement reliability of each parameter of cup positioning and HROMs. First, 10 participants (20 hips) were randomly selected from the primary data (47 participants, 94 hips). Next, two raters performed all operations, including regeneration, plane reference, preoperative planning, testing cup positioning, and testing HROMs. These two raters performed the independent data analysis 10 days apart. Finally, ICCs were analyzed by two-way analysis of variance (ANOVA). The cutoff scores for excellent reliability were set at 0.8, and the confidence level was set as 0.95 (Bonett, 2002).

Repeated measures ANOVA was used to compare the different effects on cup positioning and HROMs among four cup positioning guides, including MAG, TALG, ARLG, and IPLG. Gender and age were adjusted as covariates in the model. *Post hoc* two-sided paired *t*-tests were conducted for pairwise comparison among these 4 guides following statistically significant ANOVA omnibus tests. Significance was set at 0.05. All statistical analyses were performed using R 3.5.0^1^.

## Results

### Characteristics of Participants

Among 47 participants (94 hips), 25 (53.19%) participants were male and 22 were female (46.81%). No significant effects of gender were observed on inclination (*p* = 0.585), abduction (*p* = 0.817), extension (*p* = 0.144), flexion (*p* = 0.605), internal rotation (*p* = 0.390), and external rotation (*p* = 0.162), but significant effects were observe on anteversion (*p* = 1.01e-10) and adduction (*p* = 0.015). The average age was 47.81 ± 19.76 years with a range from 22 to 78 years. No significant effects of age were observed on abduction (*p* = 0.407), adduction (*p* = 0.059), extension (*p* = 0.055), flexion (*p* = 0.251), internal rotation (*p* = 0.533), and external rotation (*p* = 0.304), but significant effects were observe on inclination (*p* = 9.15e-4) and anteversion (*p* = 0.022). Thus, gender and age were adjusted as covariates in repeated measures ANOVA analysis.

### Good Reliability of All Measurements

**FIGURE 3:**
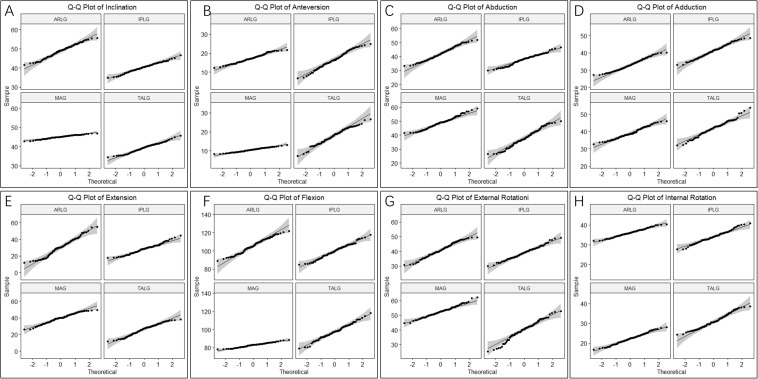
Q-Q plot of cup orientation and hip ranges of motion (HROMs). Two parameters of cup positioning, inclination **(A)** and anteversion **(B)**, and six parameters of HROMs, abduction **(C)**, adduction **(D)**, extension **(E)**, flexion **(F)**, internal rotation **(G)**, and external rotation **(H)**, were normally distributed.

**FIGURE 4:**
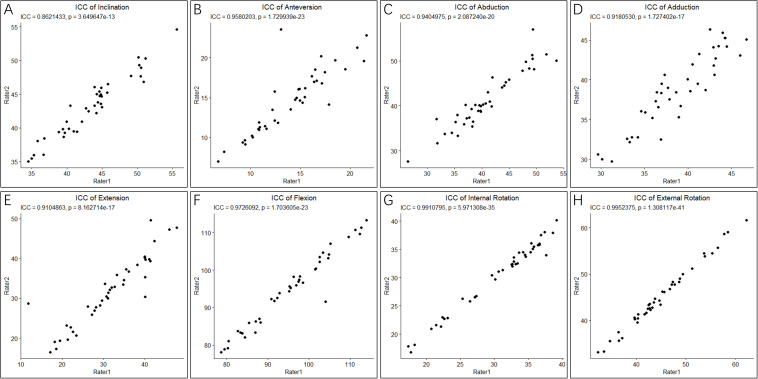
Intraclass correlation coefficients of cup orientation and hip ranges of motion (HROMs). The ICC score of two parameters of cup positioning, inclination **(A)** and anteversion **(B)**, and six parameters of HROMs, abduction **(C)**, adduction **(D)**, extension **(E)**, flexion **(F)**, internal rotation **(G)**, and external rotation **(H)**, were bigger than 0.8 with *p*-value < 0.05, suggesting that the measurements were reliable.

Shapiro-Wilk normality tests showed that all parameters for cup positioning and HROMs followed a normal distribution (*p* > 0.05) (Figure 3). The ICCs of all parameters for cup positioning and HROMs were bigger than 0.8 with *p*-value < 0.05, suggesting that the measurements were reliable (Figure 4).

### Three Personalized Guides Leading to Significantly Different Cup Positioning Compared With MAG

**FIGURE 5:**
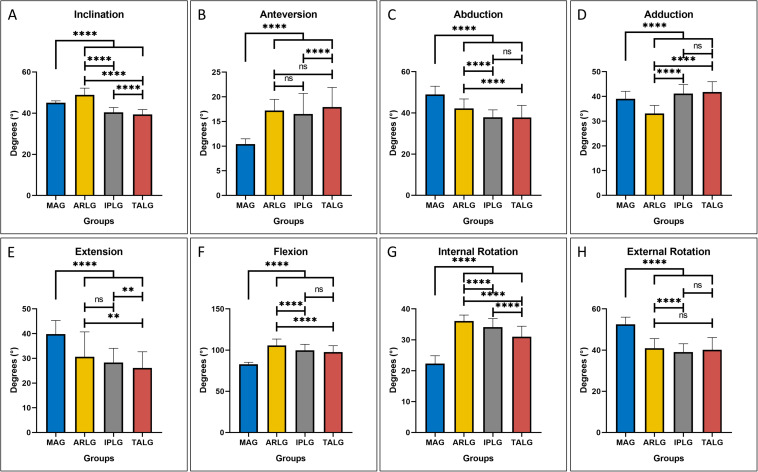
The four-cup positioning guides leading to different cup positioning and hip ranges of motion (HROMs). On the two parameters of cup positioning, great significance was observed on both inclination **(A)** and anteversion **(B)** by the four guides. All parameters of HROMs, including abduction **(C)**, adduction **(D)**, extension **(E)**, flexion **(F)**, internal rotation **(G)**, and external rotation **(H)**, were significant by the four guides. Pairwise comparison results were displayed by asterisk: ns stood for *p* > 0.05, * stood for *p* ≤ 0.05, ** stood for *p* ≤ 0.01, *** stood for *p* ≤ 0.001, and **** stood for *p* ≤ 0.0001.

The mean ± sd (standard deviation) of two parameters of cup positioning, inclination and anteversion, were displayed in Table 1. ANOVA omnibus test among 4 guides indicated that at least one guide is significantly different from others for inclination (*p* = 1.26 × 10^–101^) and anteversion (5.19 × 10^–56^), respectively. *Post hoc* pairwise comparison of inclination (Figure 5A) illustrated that compared with the use of MAG, the use of ARLG significantly increased cup inclination (*p* < 0.0001), but the use of IPLG and TALG significantly decreased cup inclination (*p* < 0.001). *Post hoc* pairwise comparison of anteversion (Figure 5B) demonstrated that the use of three personalized guides significantly increased cup anteversion compared with the use of MAG (*p* < 0.0001).

**TABLE 1:** Cup Positioning and HROMs by the four cup positioning guides.

	Cup Positioning (mean ± sd)		HROMs (mean ± sd)					
	
	Inclination (°)	Anteversion (°)	Abduction (°)	Adduction (°)	Extension (°)	Flexion (°)	Internal Rotation (°)	External Rotation (°)
MAG	45.05 ± 0.90	10.40 ± 1.07	48.91 ± 4.02	38.99 ± 3.09	39.79 ± 5.55	82.86 ± 2.35	22.30 ± 2.54	52.54 ± 3.47
ARLG	48.86 ± 3.28	17.22 ± 2.27	42.19 ± 4.57	33.11 ± 3.24	30.60 ± 10.09	105.70 ± 7.71	36.09 ± 1.92	40.94 ± 4.65
IPLG	40.40 ± 2.33	16.50 ± 4.19	37.87 ± 3.62	41.14 ± 3.66	28.30 ± 5.75	99.81 ± 7.19	34.08 ± 2.80	39.03 ± 4.05
TALG	39.36 ± 2.42	17.92 ± 3.99	37.74 ± 5.91	41.81 ± 4.15	26.13 ± 6.53	97.64 ± 7.70	30.97 ± 3.43	40.14 ± 5.95
*P*-value*	1.26 × 10^–101^	5.19 × 10^–56^	1.25 × 10^–54^	1.84 × 10^–53^	8.22 × 10^–34^	2.92 × 10^–77^	2.21 × 10^–125^	6.02 × 10^–71^

*P-value tested by ANOVA.

### Three Personalized Guides Showed Different HROMs Compared With MAG

The mean ± sd of six parameters of HROMs, which consisted of abduction, adduction, extension, flexion, internal rotation and anteversion, were displayed in Table 1. ANOVA omnibus test among four guides indicated that at least one guide is significantly different from others for abduction (*p* = 1.25 × 10^–54^), adduction (*p* = 1.84 × 10^–53^), extension (*p* = 8.22 × 10^–34^), flexion (*p* = 2.92 × 10^–77^), internal rotation (*p* = 2.21 × 10^–125^), and external rotation (*p* = 6.02 × 10^–71^), respectively.

Compared with the use of MAG, the use of the three personalized guides significantly decreased abduction (*p* < 0.0001), extension (*p* < 0.0001), and external rotation (*p* < 0.0001), respectively, illustrating that the use of three personalized guides might increase the risk of lateral impingement during abduction (Figure 5C) and increase the risk of posterior impingement and anterior dislocation during extension (Figure 5E) and external rotation (Figure 5H); the use of the three personalized guides significantly increased flexion (*p* < 0.0001) and internal rotation (*p* < 0.0001), respectively, demonstrating that the use of three personalized guides reduced the risk of anterior impingement and posterior dislocation during flexion (Figure 5F) and internal rotation (Figure 5G); the use of ARLG significantly reduced adduction (*p* < 0.0001) but the use of IPLG and TALG increased adduction (*p* < 0.0001), demonstrating that the use of IPLG and TALG reduced but the use of ARLG increased the risk of impingement and dislocation during adduction (Figure 5D).

**FIGURE 6:**
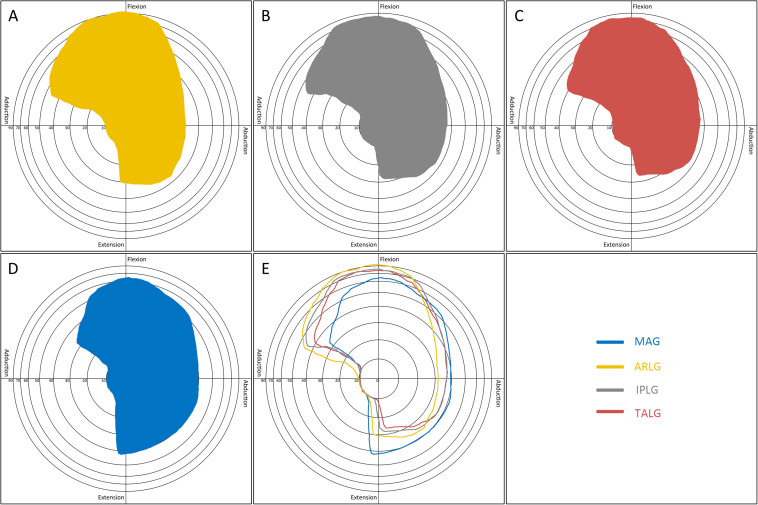
Boundary map by four cup positioning guides. The pattern in the boundary map presented a range of motion of the hip. The longitude (*X*) axis represented motions in the coronal plane: abduction (left) and adduction (right). The vertical (*Y*) axis represented motions in the sagittal plane: flexion (up) and extension (down). Concentric rings with different radiuses centered on the origin were scales indicating the degrees of circumduction, from inside to outside representing 10°, 20°, 30°, 40°, 50°, 60°, 70°, and 90°. The boundary map could reflect the effects of abduction, adduction, extension, and flexion. Acetabular rim labrum guide **(A)** improved flexion. Ischiadic-pubis line guide **(B)** and transverse acetabular ligament guide **(C)** improved adduction. Mechanical alignment guide **(D)** improved extension. By comparing the boundary maps of the four guides **(E)**, the advantage of each guide was distinct.

The boundary map reflected the combined effects of abduction, adduction, extension, and flexion. ARLG, IPLG, and TALG improved adduction and flexion but reduced extension: Flexion and adduction increased the most in the ARLG group, the least increase in the TALG group, and middle in IPLG group; the extension decreased the most in the TALG, the least in ARLG group, and the middle in IPLG group (Figures 6A–C,E). MAG significantly reduces flexion but increases extension (Figures 6D,E).

## Discussion

Our research found that the three personalized cup positioning guides, including ARLG, IPLG, and TALG, affected differently from MAG on cup positioning and HROMs. In terms of cup positioning, compared with MAG, ARLG increased (*p* < 0.0001) but IPLG and TALG decreased the inclination (*p* < 0.0001); the three personalized guides increased anteversion (*p* < 0.0001). In terms of HROMs, the three personalized guides increased flexion (*p* < 0.0001) and internal rotation (*p* < 0.0001) while reduced abduction (*p* < 0.0001), extension (*p* < 0.0001), and external rotation (*p* < 0.0001); the use of ARLG decreased (*p* < 0.0001) but the use of IPLG and TALG increased (*p* < 0.0001) adduction, compared with the use of MAG. Notably, gender had a significant effect on anteversion and adduction. Tallroth and Murtha (Tallroth and Lepistö, 2006; Murtha et al., 2008) reported that the anatomical variation between male and female pelvis was a possible cause of the difference in anteversion between males and females. Additionally, age had a significant effect on inclination and anteversion, but no previous studies have reported the role of age in cup positioning. In our study, gender and age were adjusted as covariates in repeated measures ANOVA analysis.

The inclination and anteversion are two crucial parameters to stable cups. It has been reported that changing the inclination or anteversion affected some parameters of HROMs (Chandler et al., 1982; Sonntag et al., 2017): the increasing inclination correlates with the increase of abduction and the decrease of adduction; the increasing anteversion correlates with the increase of flexion and internal rotation and decrease of extension and external rotation. Increment of flexion, adduction, and internal rotation suggests that possibility of anterior impingement and posterior dislocation reduces; increment of extension and external rotation suggests that possibility of posterior impingement and anterior dislocation reduces (Mccarthy et al., 2016). Abduction might have little effect on component dislocation, but excessive large abduction may increase hip wear, while excessive small abduction may cause impingement: excessive large or small abduction might reduce the life of the components (Patil et al., 2003; Korduba et al., 2014). Conclusively, impingement and dislocation are associated with parts of excessive small parameters of HROMs that are determined by inclination and anteversion of cups. The ways to study HROMs include cadaver study, CT three-dimension reconstruction, and skeletal muscle model (Martin et al., 2008; Mccarthy et al., 2016, 2017; Ezquerra et al., 2017; Goh et al., 2017; Jamari et al., 2017; Ohmori et al., 2017; Sonntag et al., 2017). Using cadaveric hip, Martin (Martin et al., 2008) demonstrated not only the impingement between bones or prostheses but also the limitations among ligaments, muscle, and capsule, which reflected the real situation of dislocation. However, for various reasons, few corpses could be obtained, making the study less repeatable. The CT three-dimension reconstruction, the most commonly used model, used CT data to restore pelvis, femurs, and hip joints. Researchers not only operate simulated THA with preoperative planning but also predict HROMs through simple finite element analysis (Sonntag et al., 2017). This method is highly repeatable but lacks the effect of soft tissue. The skeletal muscle model reconstructs both bones and soft tissues, such as joint capsules, muscles, and ligaments (Goh et al., 2017). It can predict the role of soft tissue in hip dislocation, but the soft tissue assessment by finite element analysis is very complicated. This study, based on CT three-dimension reconstruction, performed simulated THA through preoperative planning to study the effects of personalized guides on cup positioning and HROMs.

Preoperative planning is the most critical step in robotic assisted THA because planning errors may relate to hip dislocation. Many THA robots, such as MAKO, use MAG as a cup positioning guide (Tarwala and Dorr, 2011; Banerjee et al., 2016). However, everyone’s acetabulum is different, and even two acetabula of an individual differ from each other. Personalized cup positioning guides help orthopedists to find the most appropriate cup position. Most orthopedic surgeons choose the posterolateral approach in THA, resulting in an increased risk for posterior dislocation (Jolles et al., 2002). All personalized guides provided larger flexion and internal rotation in our research. Both IPLG and TALG provided larger adduction, indicating a lower possibility of anterior impingement and posterior dislocation.

The surgical approach is one of the important factors affecting cup positioning. The most commonly used approach is the posterolateral approach, which is easy to learn and operate but has the potential weakness of posterior hip dislocation (Tsai et al., 2008). The use of TALG, ARLG, and IPLG in preoperative planning of robotic assisted THA under the posterolateral approach might substantially reduce the incidence of posterior dislocation, through the increment of flexion, adduction, and internal rotation (Peng et al., 2019).

Among the three personalized guides, ARLG uses labrum, and TALG uses transverse acetabular ligament as reference marks. These marks may be indistinguishable from muscles in CT, and 19 (of 90) participants were excluded in this study because of the inability to identify the labrum or transverse acetabular ligament. IPLG is a less commonly used reference in traditional THA because of over-damage of soft tissue. However, the preoperative planning eliminates limitations of IPLG by a regenerated CT model that provides a fully visible bone landmark: no participants were excluded because of unidentifiable bony landmarks. Although ARLG, IPLG, and TALG may decrease the risk of posterior dislocation after robotic assisted THA, the use of IPLG was more identifiable and reliable than the other guides.

This study has some limitations. First, capsule and soft tissues are important factors limiting HROMs, and the current study does not investigate them since they do not present a clear image in CT. Future studies are warranted to establish a more accurate model with the effects of capsule and soft tissues incorporated, which may lead to a better cup positioning. Second, besides orientation that influences ROMs, other potential confounding factors such as the diameter of the cup, head, and neck (Delay et al., 2016), should be addressed by future studies. Third, although we found that the use of personalized cup positioning guides in THA could achieve better motions of flexion, adduction, and internal rotation, we do not know the effect of very small mispositions of either abduction or anteversion on HROMs, which warrant further investigation.

## Conclusion

This study evaluated the effects of MAG and three personalized guides on cup positioning and HROMs. Personalized guides provided larger flexion and internal rotation than MAG, which may reduce the risk of posterior dislocation. Among the three personalized guides, based on their stability, operability, and performance on HROM, IPLG might be the most reliable one for the preoperative planning of robotic assisted THA.

## Data Availability Statement

All datasets presented in this study are included in the article/supplementary material.

## Ethics Statement

The studies involving human participants were reviewed and approved by Ethics Committee of Tongji Medical College, Huazhong University of Science and Technology (No. IORG0003571). Written informed consent for participation was not required for this study in accordance with the national legislation and the institutional requirements.

## Author Contributions

RW and WX designed the study. XZ guided the data analysis. RW and TX helped with data analysis. RW drafted the manuscript. XZ, TX, and WX reviewed and edited the manuscript. SG collected CT data of all participants. RW and SL measured cup positioning and HROMs. SG and LH evaluated ICC. SY provided the solutions for personalized cup position guides in robotic assisted total hip arthroplasty. All authors contributed to the article and approved the submitted version.

## Conflict of Interest

The authors declare that the research was conducted in the absence of any commercial or financial relationships that could be construed as a potential conflict of interest.

## Footnotes

1 https://cran.r-project.org/

## References

[B1] Abdel M. P. Von Roth P. Jennings M. T. Hanssen A. D. Pagnano M. W. (2015). What Safe Zone? The vast majority of dislocated thas are within the lewinnek safe zone for acetabular component position. *Clin. Orthop. Relat. Res.* 474 386–391. 10.1007/s11999-015-4432-5 26150264 PMC4709312

[B2] Akiyama K. Shibuya T. (2018). Influence of femoral bowing on range of motion after total hip arthroplasty. *Int. Orthop.* 42 1795–1802. 10.1007/s00264-017-3732-7 29275431

[B3] Archbold H. A. Mockford B. Molloy D. Mcconway J. Ogonda L. Beverland D. (2006). The transverse acetabular ligament: an aid to orientation of the acetabular component during primary total hip replacement: a preliminary study of 1000 cases investigating postoperative stability. *J. Bone Joint Surg. Br.* 88 883–886. 10.1302/0301-620x.88b7.17577 16798989

[B4] Banerjee S. Cherian J. J. Elmallah R. K. Pierce T. P. Jauregui J. J. Mont M. A. (2016). Robot-assisted total hip arthroplasty. *Expert. Rev. Med. Devices* 13 47–56. 26592900 10.1586/17434440.2016.1124018

[B5] Beverland D. E. O’neill C. K. Rutherford M. Molloy D. Hill J. C. (2016). Placement of the acetabular component. *Bone Joint J.* 98-b 37–43. 10.1302/0301-620x.98b1.36343 26733639

[B6] Bonett D. G. (2002). Sample size requirements for estimating intraclass correlations with desired precision. *Stat. Med.* 21 1331–1335. 10.1002/sim.1108 12111881

[B7] Bosker B. H. Verheyen C. C. Horstmann W. G. Tulp N. J. (2007). Poor accuracy of freehand cup positioning during total hip arthroplasty. *Arch. Orthop. Trauma Surg.* 127 375–379. 10.1007/s00402-007-0294-y 17297597 PMC1914284

[B8] Chandler D. R. Glousman R. Hull D. Mcguire P. J. Kim I. S. Clarke I. C. (1982). Prosthetic hip range of motion and impingement. The effects of head and neck geometry. *Clin. Orthop. Relat. Res.* 166 284–291. 7083681

[B9] Daines B. K. Dennis D. A. (2012). The importance of acetabular component position in total hip arthroplasty. *Orthop. Clin. North Am.* 43 e23–e34. 10.1016/j.ocl.2012.08.002 23102418

[B10] De Martino I. D’Apolito R. Soranoglou V. G. Poultsides L. A. Sculco P. K. Sculco T. P. (2017). Dislocation following total hip arthroplasty using dual mobility acetabular components. *Bone Joint J.* 99-B 18–24. 10.1302/0301-620x.99b1.bjj-2016-0398.r1 28042114

[B11] Delay C. Putman S. Dereudre G. Girard J. Lancelier-Bariatinsky V. Drumez E. (2016). Is there any range-of-motion advantage to using bearings larger than 36mm in primary hip arthroplasty: a case-control study comparing 36-mm and large-diameter heads. *Orthop. Traumatol. Surg. Res.* 102 735–740. 10.1016/j.otsr.2016.04.002 27184931

[B12] Digioia A. M. III Jaramaz B. Plakseychuk A. Y. Moody J. E. Jr. Nikou C. Labarca R. S. (2002). Comparison of a mechanical acetabular alignment guide with computer placement of the socket. *J. Arthroplasty* 17 359–364. 10.1054/arth.2002.30411 11938515

[B13] Ezquerra L. Quilez M. P. Perez M. A. Albareda J. Seral B. (2017). Range of Movement for Impingement and Dislocation Avoidance in Total Hip Replacement Predicted by Finite Element Model. *J. Med. Biol. Eng.* 37 26–34. 10.1007/s40846-016-0210-4 28286463 PMC5325855

[B14] Fischer M. C. M. Eschweiler J. Schick F. Asseln M. Damm P. Radermacher K. (2018). Patient-specific musculoskeletal modeling of the hip joint for preoperative planning of total hip arthroplasty: a validation study based on in vivo measurements. *PLoS One* 13:e0195376. 10.1371/journal.pone.0195376 29649235 PMC5896969

[B15] Fukui K. Kaneuji A. Sugimori T. Ichiseki T. Matsumoto T. (2013). How far above the true anatomic position can the acetabular cup be placed in total hip arthroplasty? *Hip Int.* 23 129–134. 10.5301/hipint.5000010 23543468

[B16] Goh C. Blanchard M. L. Crompton R. H. Gunther M. M. Macaulay S. Bates K. T. (2017). A 3D musculoskeletal model of the western lowland gorilla hind limb: moment arms and torque of the hip, knee and ankle. *J. Anat.* 231 568–584. 10.1111/joa.12651 28718217 PMC5603783

[B17] Higgins S. W. Spratley E. M. Boe R. A. Hayes C. W. Jiranek W. A. Wayne J. S. (2014). A novel approach for determining three-dimensional acetabular orientation: results from two hundred subjects. *J. Bone Joint Surg. Am.* 96 1776–1784. 10.2106/jbjs.l.01141 25378504

[B18] Illgen R. L. Bukowski B. R. Abiola R. Anderson P. Chughtai M. Khlopas A. (2017). Robotic-assisted total hip arthroplasty: outcomes at minimum two-year follow-up. *Surg. Technol. Int.* 30 365–372. 28537647

[B19] Jacofsky D. J. Allen M. (2016). Robotics in arthroplasty: a comprehensive review. *J. Arthroplasty* 31 2353–2363. 10.1016/j.arth.2016.05.026 27325369

[B20] Jamari J. Anwar I. B. Saputra E. Van Der Heide E. (2017). Range of motion simulation of hip joint movement during salat activity. *J. Arthroplasty* 32 2898–2904. 10.1016/j.arth.2017.03.056 28499625

[B21] James W. Kirk J. Werner H. Jens R. Gordon F. Flávio T. (2010). Orthopaedic practice in total hip arthroplasty and total knee arthroplasty: results from the Global Orthopaedic Registry (GLORY). *Am. J. Orthop.* 39 5–13. 21290026

[B22] Jie W. Ling B. Jianwen W. Kai L. Rong L. Xiaojie Z. (2016). [Range of Motion of Shoulder and Hip in Chinese Han Population and Its Influence Factors:Focus on Gender and Age]. *Chin. J. Forensic Med.* 31 488–492.

[B23] Jolles B. M. Zangger P. Leyvraz P. F. (2002). Factors predisposing to dislocation after primary total hip arthroplasty. *J. Arthroplasty* 17 282–288. 10.1054/arth.2002.30286 11938502

[B24] Jones S. A. (2015). The prevention and treatment of dislocation following total hip arthroplasty: efforts to date and future strategies. *Hip Int.* 25 388–392. 10.5301/hipint.5000273 26044529

[B25] Korduba L. A. Essner A. Pivec R. Lancin P. Mont M. A. Wang A. (2014). Effect of acetabular cup abduction angle on wear of ultrahigh-molecular-weight polyethylene in hip simulator testing. *Am. J. Orthop.* 43 466–471. 25303445

[B26] Martin H. D. Savage A. Braly B. A. Palmer I. J. Beall D. P. Kelly B. (2008). The function of the hip capsular ligaments: a quantitative report. *Arthroscopy* 24 188–195. 10.1016/j.arthro.2007.08.024 18237703

[B27] Mccarthy T. F. Alipit V. Nevelos J. Elmallah R. K. Mont M. A. (2016). Acetabular cup anteversion and inclination in hip range of motion to impingement. *J. Arthroplasty* 31 264–268. 10.1016/j.arth.2016.01.067 27067753

[B28] Mccarthy T. F. Nevelos J. Elmallah R. K. Chughtai M. Khlopas A. Alipit V. (2017). The effect of pelvic tilt and femoral head size on hip range-of-motion to impingement. *J. Arthroplasty* 32 3544–3549. 10.1016/j.arth.2017.06.016 28712801

[B29] Murray D. W. (1993). The definition and measurement of acetabular orientation. *J. Bone Joint Surg. Br.* 75 228–232. 10.1302/0301-620x.75b2.8444942 8444942

[B30] Murtha P. E. Hafez M. A. Jaramaz B. Digioia A. M. (2008). Variations in acetabular anatomy with reference to total hip replacement. *J. Bone Joint Surg. Br.* 90 308–313. 10.1302/0301-620x.90b3.19548 18310751

[B31] Ohmori T. Kabata T. Kajino Y. Taga T. Hasegawa K. Inoue D. (2017). Differences in range of motion with the same combined anteversion after total hip arthroplasty. *Int. Orthop.* 42 1021–1028. 10.1007/s00264-017-3653-5 28990125

[B32] Patil S. Bergula A. Chen P. C. Colwell C. W. Jr. D’lima D. D. (2003). Polyethylene wear and acetabular component orientation. *J. Bone Joint Surg. Am.* 85-A(Suppl. 4), 56–63. 10.2106/00004623-200300004-00007 14652394

[B33] Peng Y. Arauz P. Desai P. Byers A. Klemt C. Kwon Y. M. (2019). In vivo kinematic analysis of patients with robotic-assisted total hip arthroplasty during gait at 1-year follow-up. *Int. J. Med. Robot.* 15:e2021. 10.1002/rcs.2021 31144768

[B34] Peter W. F. Dekker J. Tilbury C. Tordoir R. L. Verdegaal S. H. Onstenk R. (2015). The association between comorbidities and pain, physical function and quality of life following hip and knee arthroplasty. *Rheumatol. Int.* 35 1233–1241. 10.1007/s00296-015-3211-7 25586654 PMC4436688

[B35] Pivec R. Johnson A. J. Mears S. C. Mont M. A. (2012). Hip arthroplasty. *Lancet* 380 1768–1777. 23021846 10.1016/S0140-6736(12)60607-2

[B36] Qin J. Xu Z. Dai J. Chen D. Xu X. Song K. (2018). New technique: practical procedure of robotic arm-assisted (MAKO) total hip arthroplasty. *Ann. Transl. Med.* 6 364–364. 10.21037/atm.2018.09.30 30370291 PMC6186553

[B37] Rutherford M. O’connor J. D. Hill J. C. Beverland D. E. Lennon A. B. Dunne N. J. (2018). Patient positioning and cup orientation during total hip arthroplasty: assessment of current UK practice. *Hip Int.* 29 89–95. 10.1177/1120700018760818 29783888

[B38] Sato Y. Sasama T. Sugano N. Nakahodo K. Nishii T. Ozono K. (2000). “Intraoperative simulation and planning using a combined acetabular and femoral (caf) navigation system for total hip replacement,” in *Medical Image Computing and Computer-Assisted Intervention – MICCAI 2000*, eds Delp S. L. Digoia A. M. Jaramaz B. (Berlin: Springer), 1114–1125. 10.1007/978-3-540-40899-4_116

[B39] Scifert C. F. Noble P. C. Brown T. D. Bartz R. L. Kadakia N. Sugano N. (2001). Experimental and computational simulation of total hip arthroplasty dislocation. *Orthopedic Clin.* 32 553–567. 10.1016/s0030-5898(05)70226-1 11689369

[B40] Seagrave K. G. Troelsen A. Malchau H. Husted H. Gromov K. (2017). Acetabular cup position and risk of dislocation in primary total hip arthroplasty. *Acta Orthop.* 88 10–17. 10.1080/17453674.2016.1251255 27879150 PMC5251254

[B41] Sonntag R. Braun S. Al-Salehi L. Reinders J. Mueller U. Kretzer J. P. (2017). Three-dimensional friction measurement during hip simulation. *PLoS One* 12:e0184043. 10.1371/journal.pone.0184043 28886102 PMC5590873

[B42] Sotereanos N. G. Miller M. C. Smith B. Hube R. Sewecke J. J. Wohlrab D. (2006). Using intraoperative pelvic landmarks for acetabular component placement in total hip arthroplasty. *J. Arthroplasty* 21 832–840. 10.1016/j.arth.2005.12.001 16950035

[B43] Stansfield B. W. Nicol A. C. Paul J. P. Kelly I. G. Graichen F. Bergmann G. (2003). Direct comparison of calculated hip joint contact forces with those measured using instrumented implants. An evaluation of a three-dimensional mathematical model of the lower limb. *J. Biomech.* 36 929–936. 10.1016/s0021-9290(03)00072-1 12757801

[B44] Tallroth K. Lepistö J. (2006). Computed tomography measurement of acetabular dimensions: normal values for correction of dysplasia. *Acta Orthop.* 77 598–602. 10.1080/17453670610012665 16929436

[B45] Tarwala R. Dorr L. D. (2011). Robotic assisted total hip arthroplasty using the MAKO platform. *Curr. Rev. Musculoskelet. Med.* 4 151–156. 10.1007/s12178-011-9086-7 21728013 PMC3261258

[B46] Tezuka T. Heckmann N. D. Bodner R. J. Dorr L. D. (2019). Functional safe zone is superior to the lewinnek safe zone for total hip arthroplasty: why the lewinnek safe zone is not always predictive of stability. *J. Arthroplasty* 34 3–8. 10.1016/j.arth.2018.10.034 30454867

[B47] Tsai S. J. Wang C. T. Jiang C. C. (2008). The effect of posterior capsule repair upon post-operative hip dislocation following primary total hip arthroplasty. *BMC Musculoskelet. Disord.* 9:29. 10.1186/1471-2474-9-29 18307820 PMC2292160

[B48] Turley G. A. Williams M. A. Wellings R. M. Griffin D. R. (2013). Evaluation of range of motion restriction within the hip joint. *Med. Biol. Eng. Comput.* 51 467–477. 10.1007/s11517-012-1016-3 23263850 PMC3589629

[B49] Won S. H. Lee Y. K. Ha Y. C. Suh Y. S. Koo K. H. (2013). Improving pre-operative planning for complex total hip replacement with a Rapid Prototype model enabling surgical simulation. *Bone Joint J.* 95-B 1458–1463. 10.1302/0301-620x.95b11.31878 24151263

[B50] Zeng Y. Lai O. J. Shen B. Yang J. Zhou Z. K. Kang P. D. (2014). Three-dimensional computerized preoperative planning of total hip arthroplasty with high-riding dislocation developmental dysplasia of the hip. *Orthop. Surg.* 6 95–102. 10.1111/os.12099 24890290 PMC6583409

[B51] Zhang H. Wang Y. Ai S. Chen X. Wang L. Dai K. (2017). Three-dimensional acetabular orientation measurement in a reliable coordinate system among one hundred Chinese. *PLoS One* 12:e0172297. 10.1371/journal.pone.0172297 28207829 PMC5313188

